# Exploring native and non-native English speaker teachers’ perceptions of English teacher qualities and their students’ responses

**DOI:** 10.3389/fpsyg.2023.1175379

**Published:** 2023-08-15

**Authors:** Liwei Deng, Lawrence Jun Zhang, Naashia Mohamed

**Affiliations:** Faculty of Education and Social Work, University of Auckland, Auckland, New Zealand

**Keywords:** EFL teachers, teacher quality, misalignment, NESTs, NNESTs, perceptions

## Abstract

Due to globalization, English has gradually become a *lingua franca*, leading to a rising demand for proficient English teachers all over the globe. In China, more EFL teachers are being recruited, particularly at the tertiary level, with a greater preference for so-called “native English speaking teachers (NESTs)” over “non-native English-speaking teachers (NNESTs)” due to the impacts of native-speakerism. Research has shown NESTs, NNESTs, and students are often misaligned in terms of beliefs about language learning and teaching which affect teaching effectiveness as well as student achievement. Recognizing this issue, this study investigated NESTs’, NNESTs’, and Chinese English-major students’ perceptions of characteristics of effective EFL teachers at four mid-tier universities across China. Findings from semi-structured interviews with 16 students suggest that NNESTs and Chinese English-major students had similar views on language learning and teaching. Both groups valued prerequisite qualities such as having expert knowledge, language skills, teaching skills, and professionalism. NESTs, however, valued qualities such as caring, patience, flexibility, engagement, and awareness of students’ learning needs. These differences are likely the result of these two groups of teachers’ linguistic, cultural, and educational background differences. The highly uniform views of the two groups of teachers suggest that they tended to emphasize certain qualities while disregarding others. These findings suggest the need to raise teachers’ and students’ awareness of the benefits of different types of teacher qualities so that curriculum design and lesson planning can be implemented for better instructional alignment to ultimately improve teaching effectiveness.

## Introduction

English has gradually become a *lingua franca* and is used among speakers of different first languages worldwide (Fang, 2016; Llurda and Calvet-Terré, 2022). It plays vital roles in various fields such as scientific publications, international trade, and mass international telecommunications (Yang and Zhang, 2015; Rao, 2019). As a result, an increasing number of countries are making English proficiency a national policy goal (Taguchi, 2014; Hennebry-Leung and Gao, 2022; Shan and Xu, 2022). This potentially has contributed to the drastic increase in the number of English learners worldwide, with an estimation of over 1.5 billion (Szimigiera, 2021). For instance, China has made English a compulsory subject from Primary Three (age 9 or 10 years) onwards, and it is estimated that there are more than 400 million English learners in China (Galloway, 2017).

This expansion of the global English education market has created a growing demand for effective English as a second language (ESL) and English as a foreign language (EFL) teachers. In the field of English language teaching (ELT), the majority of teachers are multilinguals who come from language backgrounds other than English. These so-called “non-native English speaking teachers” (NNESTs) constitute about 80% of the teaching force globally (Moussu, 2018). Nevertheless, in many ESL/EFL contexts, there is a tremendous demand for English monolingual teachers—often referred to as “native English-speaking teachers” (NESTs), based on the unfounded myth that they are more proficient and skilled, making them superior English language teachers (Llurda and Calvet-Terré, 2022). Holliday (2018) used the term “native-speakerism” to describe this pervasive ideology and pointed out that it has been mainstream in the ELT field for decades. Historically, native-speakerism has been extensively accepted by the public, particularly by foreign language students and their teachers as well as education program administrators. It shapes a discriminatory and dichotomic perspective on the linguistic and cultural differences between NESTs and NNESTs. Even though this ideology is being contested, it remains a persistent issue in many ELT contexts, including China, legitimizing the discriminatory practices against NNESTs (Moussu, 2018; Zhang, 2021; Zhang and Zhang, 2021; Liu, 2021a).

Chinese universities have recently recruited more English teachers (especially NESTs) because of the pursuit of internationalization of higher education, the increasing demand for English as an important skill, and the expansion of English majors. Consequently, English majors became the largest undergraduate program in Chinese universities by 2013 (Dai and Wang, 2014; He, 2020; Zhang and Zhang, 2021). However, research has shown that NESTs’ views of effective teaching are often viewed as misaligned with local Chinese EFL teachers and students. For example, NESTs’ teaching styles conflicted with Chinese EFL students’ learning styles, and their preferences for writing feedback differed from local Chinese teachers (Rao, 2010; Cheng and Zhang, 2022). These findings indicate that NESTs in China have introduced teaching methods that might have succeeded in other settings but have not adapted their teaching to the students’ cultural and linguistic contexts.

Even though previous studies have investigated the different perceived positive and negative characteristics of NESTs and NNESTs (Zhang, 2004a,b; Javid, 2016; Cruz and Auman, 2018; Nomura and Mochizuki, 2018; Kiczkowiak, 2019; Wang and Fang, 2020; Zhang and Zhang, 2021; Qiu and Fang, 2022), few studies have investigated their perceptions of effective teacher qualities. In addition, most studies in China have concentrated on the response of either students or English teachers (Rao, 2010; Ma, 2016; Huang, 2017; Wang and Fang, 2020; Cheng and Zhang, 2022). The student participants often consist of non-English majors instead of English majors. Thus, this study intends to fill these gaps by investigating the perceptions of NESTs, NNESTs, and Chinese English majors of effective English teaching in the Chinese context. It aims to understand the differences between NESTs’, NNESTs’, and Chinese English majors’ perspectives on effective teachers’ characteristics. It provides implications for teachers to modify their teaching to improve instructional alignment and ultimately improve teaching effectiveness.

## Literature review

### Definition of NESTs and NNESTs

Traditionally, a native speaker of a language is defined as someone who has been speaking the language as their first language since birth and raised in that language (Chomsky, 1965). The native speaker was assumed to possess a perfect mastery of the language. In comparison, a non-native speaker is described as someone who learns the target language as a second or foreign language. This classification of native and non-native speakers has received heavy criticism in recent decades because of its underlying raciolinguistic ideologies (Cheng et al., 2021). For instance, Braine (2010, p. 9) observed that “the term ‘native speaker’ undoubtedly has positive connotations: it denotes a birthright, fluency, cultural affinity, and sociolinguistic competence,” and “the term ‘non-native speaker’ carries the burden of the minority, of marginalization and stigmatization.” However, some researchers have viewed NNESTs labeling as a double-edged blade (Selvi, 2014). Even though using the NNEST label could have certain disadvantages such as debasing, othering NNESTs, and referring to a false standard (i.e., native-speakerism), it is also seen by some as having some benefits. This includes valuing/admitting the periphery, making it easy to organize against discrimination, using the identification already existing in the research field, and benefitting the profession (valuing education and expertise; Selvi, 2014). Therefore, the NNESTs/NESTs classification is used to emphasize the issues in China (see also Zhang and Zhan, 2014; Zhang and Zhang, 2021; Fang et al., 2022; Sun et al., 2022). Most of the Chinese EFL institutions have adopted Kachru et al.’s (2006) classification and viewed English speakers who were born and raised in “inner-circle countries” such as the UK, the USA, Anglophone Canada, Australia, and New Zealand, and specific “outer-circle countries,” or former British colonies, such as South Africa and Singapore as native English speakers. Expat English teachers who belong to this group are regarded as NESTs. Although many expats who are regarded as “NNESTs” are eligible EFL teachers, Chinese institutions are prone to hire NESTs and local Chinese English speaker teachers who are born and raised in China. While we recognize the raciolinguistic nature of this dichotomy, we use these terms in this paper as the study we present compares NESTs’ and NNESTs’ perceptions of English teacher qualities and their students’ responses. Such a comparison is significant for colleagues in the English teaching profession and administrators when working with teachers of different backgrounds in Chinese universities.

### Misalignments between NESTs, NNESTs, and students on effective teaching

Cohen (1987) proposed the term “instructional alignment “and postulated that it is required across three systems (instructional processes, intended outcomes, and assessment) for effective learning. Specifically, teachers need to align how they teach, what they want students to learn, and what is assessed to teach effectively. In this study, the term “alignment” indicates teachers’ instructional processes, intended outcomes, and assessments in accordance with students’ demands and interests. In contrast, misalignments between teachers and students can cause problems such as students feeling frustrated that teachers fail to meet their needs and affect students’ learning outcomes and their evaluations of teachers (Rao, 2010; Yang, 2018; Cheng and Zhang, 2022).

Today, most EFL teachers in China are still indigenous Chinese EFL teachers (Pawan, 2017; Zhang, 2021; Zhang and Cheng, 2021). Nevertheless, with the growth of the number of NESTs in China, the constitution of the faculty has become more varied. This transformation has led to new challenges and increased the existing ones for effective ELT in China. Despite that NESTs are deemed as more effective teachers (Wang and Fang, 2020), research has indicated NESTs’ perceptions of effective teaching are often perceived as misaligned with local Chinese EFL teachers and students. For example, Rao (2010) interviewed 20 Chinese university students and found that NESTs’ teaching styles were misaligned with Chinese EFL students’ learning styles. The students were unaccustomed to NESTs’ hands-on style of performing role-plays and games which made it difficult for students to focus on the curriculum requirements. They were also dissatisfied with NESTs’ “open” style, as they would provide several correct answers for a given question. Furthermore, the students were unused to NESTs’ “intuitive-random” style as their manners differed from the traditional Chinese figure that teachers should behave like authorities. Moreover, the students criticized NESTs for favoring a “global” teaching style and used holistic strategies such as guessing and inferencing for searching the main idea of reading and listening materials and for concentrating less on the analysis of linguistic details. The fact is that the students preferred a more analytical style for studying complex linguistic styles, structures, and rhetoric. Lastly, NESTs were viewed to be insensitive to students’ linguistic difficulties and less capable of offering students with local examples. These findings suggest that NESTs in China have imported teaching approaches that may have worked in other contexts, but have not adapted their teaching in response to the students’ language and cultural backgrounds.

Cheng and Zhang (2022) observed that NESTs and NNESTs in the Chinese EFL university setting held different beliefs in various aspects of writing feedback scope (i.e., the extent of the feedback teachers should provide their students). For instance, most NESTs favored focused feedback and indicated that teachers should address only a few types of errors and overlook the others; NNESTs, however, preferred comprehensive feedback and argued that EFL learners usually could not recognize global issues autonomously and lacked sufficient English writing rhetorical understanding.

The misalignment also occurs between NNESTs and students in their attitudes and beliefs toward effective teaching (Yang, 2018; Saleem et al., 2022). Yang (2018) also found divergences between NNESTs’ and Chinese EFL students’ beliefs regarding instruction. For example, one of the teacher participants preferred a bottom-up approach to reading instruction, believing that sentence structure and vocabulary were fundamental, while five out of the six student participants leaned toward the top-down approach since it provided context and direction for reading. The teacher participant also favored a more teacher-centered model while some students required more autonomy in learning. Moreover, misalignments can arise between teachers’ beliefs and practices (Zhang and Said, 2014; Bai and Yuan, 2019; Zhao and Zhang, 2022; Zhang and Sun, 2022).

The misalignments between teachers’ and students’ perceptions concerning effective teaching often harm students’ learning results and their assessments of teachers. For example, teachers could meet difficulties understanding students’ overall needs (Lo, 2015; Meissel et al., 2017; Ranjit, 2022), which makes students underperform, feel dissatisfied with their abilities, or be disappointed with certain parts of the class (Rao, 2010; Roothooft and Breeze, 2016).

### Effective teachers’ characteristics

There are disagreements in the beliefs of students and teachers about the characteristics of effective teachers, as it is a complex concept that has yet to reach consensus in both general education and more specialized settings such as English education (Farrell, 2015; Stronge, 2018). However, research has often suggested that certain qualities such as caring are essential for successful English language teaching (Huang, 2017; Tatipang et al., 2022). Characteristics of effective teachers are commonly categorized into general and subject-specific characteristics. The nature of education and the commonalities across various disciplines determine that some of these effective characteristics can be broadly applicable and play an important role. These qualities are regarded as prerequisites or key characteristics. For example, Stronge (2018, p. 18) outlined some important characteristics from research literature: verbal ability, knowledge of teaching and learning, certification status, content knowledge, and teaching experience. In addition to characteristics related to teaching and management procedures, affective aspects can have a positive psychological impact on students and are viewed by some researchers as equally important in terms of student success (Stronge, 2018; Zhang et al., 2019).

Affective characteristics such as fairness, sensitivity to students’ needs, and caring are often associated with effective English teachers (Huang, 2017). For example, Tatipang et al. (2022) investigated the qualities of effective English teachers recognized by EFL Students in Indonesia. A total of 120 students from grade 11 in science, social, and Language class participated in the study. The most desirable characteristics include the use of easy English language to help students understand, assess what students have learned rationally, control themselves and not get angry, and know and master English vocabulary well. Research has often considered the perspectives of students and teachers as both are valuable sources of information that can provide us with a better understanding of the issue. Students have the advantage of being exposed to teachers with different characteristics and teaching practices and are consequently able to evaluate which characteristics are effective. Teachers, on the other hand, have the benefit of being former students and have gone through training, undertaken teaching activities, and learned from other teachers (Lavin et al., 2012). This has given them a comprehensive understanding of what makes a good teacher. A few studies compared the perspectives of NESTs and NNESTs on effective teaching in China (Rao, 2010; Ma, 2016; Huang, 2017; Wang and Fang, 2020), and mostly covered the views of NNESTs and non-English majors, yet the opinions of NESTs and Chinese English majors have not been sufficiently explored. Research has clearly shown NESTs have a different understanding from local NNESTs and students, and English majors also differ from non-English majors in terms of their understanding of English teachers (Rao, 2010; Cheng and Zhang, 2022). Therefore, it is necessary to compare and analyze the opinions of the participants in the study (NESTs, NNESTs, and English majors) on teaching effectiveness to offer us valuable information and a more comprehensive view of the issues. Specifically, this study addressed the following research questions:

1. What are the native and non-native English speaker teachers’ and their Chinese students’ perceptions of effective English teacher qualities?
2. How do their perceptions differ?

### Conceptual framework

Based on the literature review and to address the research questions stated above, this study adopted a conceptual framework that consisted of three main concepts: native-speakerism (Holliday, 2018), instructional (mis)alignment (Cohen, 1987), and effective teachers’ characteristics (Stronge, 2018). These concepts helped to explain and explore the perceptions of effective teaching among students, NESTs, and NNESTs in EFL contexts. They would also be useful frames of reference for our interpretation of the potential misalignment between perceptions and actual pedagogical practices, especially when we intended to examine the implications of these perceptions for improving language teaching. Ultimately, these findings are expected to help us deepen our understanding of what it means when teaching effectiveness becomes a key area of focus.

## Methods

### Qualitative research design

We adopted a generic qualitative design, which gave us more flexibility, creativity, and responsiveness to deal with the research context and research questions and enabled us to capture the complexity and diversity of the phenomenon and related variables. However, a generic qualitative design also has some limitations, such as the possible lack of rigor, clarity, credibility, and generalizability of the study if not conducted properly (Queirós et al., 2017).

### Participants

The research was conducted at 4 mid-tier universities across China to better represent the perceptions of most NESTs, NNESTs, and English majors. A total of 16 students, 4 NNESTs, and 3 NESTs participated in the study. Although it was initially designed to interview 1 NEST and 1 NNEST from each university, making a total of 4 NESTs and 4 NNESTs. which would result in a more balanced number of teacher participants from each university. Most of the NESTs were abroad at the time because of the Covid-19 pandemic, and few of them were available for online interviews. As a result, three NESTs from the United States of America with English as their first language participated in the study. Two had a non-English or education-related Bachelor’s Degree and a Certificate in Teaching English to Speakers of Other Languages (TESOL), and the third one had a PhD in English Literature. In comparison, three NNESTs had PhDs in English or education-related majors and one was a PhD student at the time when the data were collected. Table 1 provides the demographic information of the student participants of the quantitative study including students’ gender, age, year of study, high school, and university.

**Table 1:** General information of student participants in the qualitative study.

		Frequency	Percent
Gender	Male	5	31.3
	Female	11	68.8
Age	19–21	12	75
	22–24	4	25
Year of study	Third-year	14	87.5
	Fourth-year	2	12.5
High school	Township	10	62.5
	City	6	37.5

Tables 2, 3 display the demographic information on the NESTs’ and NNESTs’ gender, nationality, first language, year of teaching, and the universities at which they were teaching at the time of data collection for this study.

**Table 2:** NEST participants for the qualitative study.

Questions	Responses from NEST participants		
Pseudonyms	Henry	Lucy	Claire
Gender	Male	Female	Female
Nationality	The USA	USA	USA
First language	English	English	English
Year of teaching	3–4 years for all NEST participants		
University	A	B	C

**Table 3:** NNEST participants for the qualitative study.

Questions	Responses from NNESTs participants			
Pseudonyms	John	David	Nora	Lily
Gender	Male	Male	Female	Female
Nationality	China	China	China	China
First language	Chinese	Chinese	Chinese	Chinese
Year of teaching	7–27 years for all NNEST participants			
University	A	B	C	D

The study employed convenience sampling as a result of the restrictions imposed by the Covid-19 pandemic, as our access to potential participants was limited and the participants had limited availability. The universities involved in this study adopted the national English syllabus for English majors, *the Syllabus for English Majors in Institutions of Higher Education* (Ministry of Education of China, 2000) for the prevision of English major education, and the students were enrolled in four-year degree programs (He, 2020). The age of participants ranged from 19 to 24, with more than 10 years of English learning experience from the third grade of primary school to high school. I selected juniors and seniors as the participants of this study since they had over 3 years of English learning experience and had undertaken courses taught by both NESTs and NNESTs at the university. The teacher participants were chosen based on their availability and teaching experiences, all of them had taught English for more than 2 years. Experienced teachers differed from novice teachers in time, teaching experiences, and classroom practice. They were more knowledgeable about the education system, students, and teaching material (Stronge, 2018), thus making them ideal for interviewing to gain insight into teaching effectiveness.

### Instruments and data collection

To better understand students’ and teachers’ perceptions of teaching effectiveness, three semi-structured interview guides were designed. Students, NESTs, and NNESTs were asked to explain their perceptions of teaching effectiveness. For Chinese teachers and students, the interviews were translated into their native language (Chinese). The semi-structured interview guides contained main questions and follow-up sub-questions. The main questions encompassed the main content of the research questions and contained questions for all participants. The main questions acted as warm-up questions to familiarize participants with the subject and create a relaxing atmosphere. The follow-up sub-questions were intended to enhance the flow of the interview process and to gain more precise and detailed answers from the participants. They were selectively asked based on different participants’ responses. The students were asked about their views of the effective qualities of English teachers, while the teachers were asked about their background, teaching experience, and opinions on the qualities of effective teachers. The interviews for students took 7 h and 24 min, with most of them lasting between 10 and 25 min. The interviews for teachers took 3 h and 39 min, with a majority of them being between 18 and 58 min.

To minimize the limitations of using qualitative data collection tools, we took measures to ensure the quality and rigor of the research process. We recruited a sufficient number of participants from different groups (students, NESTs, and NNESTs) to achieve representativeness and drew up our interview questions from the reviewed literature (Rao, 2010; Huang, 2017; Stronge, 2018) and the conceptual framework (Cohen, 1987; Holliday, 2018; Stronge, 2018) to ensure that they were relevant and valid for the research questions and objectives. For instance, Huang (2017) asked non-English majors’ perceptions of NESTs, NNESTs, and their English accents, and some of their questions provided inspirations for the current study. Also, we conducted a pilot study with two students, two NESTs and two NNESTs to validate the interview questions.

#### Semi-structured interview guides for teachers

The interview guides for both native and non-native English-speaking teachers consist of the same questions. The teachers were asked about their linguistic, educational, and cultural background, which includes their nationality, first language, and experience of learning other languages. They were also asked about their English teaching experience, especially in China. The teachers were asked to explain their understanding of the qualities of an effective teacher, their teaching philosophies and approaches, and the formation of these philosophies and approaches. These questions aimed to understand the identities of the participants and how teachers’ beliefs of effective teachers’ qualities have been formed and whether their teaching practices correspond to their beliefs, providing valuable information for further interpreting their perceptions.

#### Semi-structured interview guide for students

The interview guide for students was adjusted from those for teachers. The students were asked to explain their perceptions of the characteristics of an effective teacher, describe the best English teacher they had, and their best qualities. These questions aimed to understand students’ beliefs of effective teachers’ qualities and the formation of these beliefs. In addition, the students were asked about their perceptions of whether “nativeness” is a precondition for being an effective teacher, and the characteristics of native and non-native English-speaking teachers to understand the impact of “native-speakerism” on the students.

### Data analysis

The semi-structured interviews were audio-recorded and transcribed by the researchers. The transcripts were checked for accuracy and anonymized, with the names of the participants being presented with pseudonyms. The qualitative data from the semi-structured interviews were analyzed using thematic analysis following Braun and Clarke’s (2012) six-step process: (1) familiarization: the researchers immersed themselves in the data by listing to audio recordings, noting words of interest, thoroughly reading the data, and critically considering the meaning of the data; (2) generating initial codes: the transcripts were imported into NVivo 11 for Windows, the transcripts were coded line by line, the researchers assigned labels to segments of text to represent meaningful units of information. The codes were generated and modified as codes to capture the variety and patterns found within the data. For instance, an NNEST said “In terms of teaching, we are more compact, and also being able to know what the students want…” and it was coded as “perceived positive characteristics of NNESTs”; (3) searching for themes: the researchers examined the coded data to identify similarities and overlapping areas between codes, and group codes that were similar to create themes and subthemes, For example, the generated codes such as “(perceived) positive characteristics of NNESTs,” and “(perceived) positive characteristics of NESTs” were found in three groups and they formed the theme “(perceived) differences between NESTs and NNETs”; (4) reviewing potential themes: the researchers recursively reviewed the generated themes to further refine and clarify them, and ensured that these themes related to the research question and objectives, the literature review, and the conceptual framework. (5) defining and naming themes: the researchers summarized the essence and clearly described the unique qualities of each theme (see Appendices A,B) and and (6) producing the report: the researchers analyzed and synthesized the results, compared and contrasted the perspectives of different groups of participants, discussed the implications and limitations of the study, and presented them in the following sections.

## Findings and discussion

The main findings of this study are based on the thematic analysis of 23 semi-structured interviews with teachers and students. The generated themes from the thematic analysis of this section are presented in Table 4.

**Table 4:** Themes, codes, descriptions, and examples for effective teachers’ qualities.

Theme	Code	Description and example
Effective teaching	Effective teachers’ qualities and behaviors	Referring to teachers’ qualities and behaviors that are regarded by learners to have a positive effect on learning outcomes.
		e.g.*, Firstly, he should have a high level of professionalism. Secondly, he should be responsible. (Jennifer-FS)*
	Expectation of teaching	Learners’ idealized teaching form and environment.
		e.g.*, The expectations were that teachers would be able to show us more about the Western world, different from the Chinese model (Robert-MS)*
	Suggestions on improving teaching effectiveness	Learners’ suggestions on improving the teaching effectiveness of English classes.
		e.g.*, I think this is something that needs to be improved. I think I would like to learn more about culture from NESTs (Isabella-FS)*

In general, the students and NNESTs had more resemblances than the NESTs about effective teaching. Both emphasize teachers’ teaching abilities, language abilities, and professionalism, and viewed knowledge as one of the most essential qualities. However, there were slight differences. The students paid more attention to personal qualities such as being confident, passionate, inclusive, humorous, strict, and having good pronunciation. On the other hand, the NNESTs were more concerned with understanding students’ psychological development and learning processes and using theoretical knowledge to help students. The following conversation extracts from Ryan and John illustrate the typical students’ and NNESTs’ thoughts on effective teachers’ qualities. The source from the male student Ryan is coded as (Ryan-MS). For Ryan, the most crucial qualities for teachers are having good knowledge, teaching skills, and being able to guide students and give feedback.

Ryan: Most importantly, an effective English teacher should be knowledgeable in many areas. English is a language, and language involves many areas, and the teacher should have a basic knowledge of each area. I think that having good speaking skills is an aspect that can attract the interest of students. Apart from these two requirements, a qualified teacher, he/she should have very solid teaching skills and be able to guide you on the academic side and give you constructive advice. (Ryan-MS)

For John, the most important requirements for teachers are also having good knowledge and teaching skills. He also stated that teachers must have a strong understanding of students’ psychology and learning process.

John: I think the first and foremost thing is strong professional knowledge. For example, when you teach a class, your level of professionalism for teaching this class is definitely the most important. The second is your ability to teach. You have to be able to teach and know how to teach as a teacher… In addition, you need to understand their psychological development process. (John-MNNEST)

In comparison, the NESTs rarely brought up the qualities that Chinese students and NNESTs highly valued such as knowledge, teaching skills, and professionalism. They put more emphasis on affective qualities and teacher-student relationships such as caring, patience, flexibility, engagement, and being aware of students’ learning needs. Both Lucy and Clare placed great importance on affective qualities.

Lucy: I think you have to be patient. You have to be caring for your students. You have to be able to be flexible. I think you should be, I'm not sure, I guess that's it. Be flexible. Be caring. (Lucy-FNEST)

Clare: You need to be engaging. Chinese students have a hard time participating in class, so you need to be able to get them to volunteer and try because Chinese students have a hard time getting an answer wrong or speaking if they're not perfect. But you can't practice if you're afraid of never being imperfect. So you have to be very engaging with Chinese students. You have to have good pronunciation, and you have to be patient and have a good grasp of English in general, and know a lot of the grammatical rules of English. (Clare-FNEST)

The NESTs and the NESTs also significantly differed in their teaching philosophies and approaches. The NESTs had a more humanistic view and emphasized a positive teacher-student relationship and mutual engagement in class. For example, one of the NESTs, Henry, compared teaching to having a journey with students, and they were all involved in this labor of love. On the other hand, the NNESTs were more practical. They preferred to talk about establishing solid knowledge and abilities for students, giving out homework, and preparing for students’ future careers.

Overall, Chinese teachers and students hold more similar views on effective teachers’ characteristics than NESTs. Both NNESTs and Chinese learners emphasize pedagogical aspects instead of affective characteristics, while NESTs valued affective qualities more than educational ones. These findings correspond to prior investigations conducted in the Chinese setting (Rao, 2010; Ma, 2016; Huang, 2017; Sarwal and Lamb, 2018), which revealed that both students and NNESTs give great importance to essential characteristics such as knowledge, language abilities, instruction abilities, and professionalism. Research in general education also suggested that teachers, students, and university directors strongly prioritize subject knowledge (Stronge, 2018).

This is likely because the vast majority of EFL teachers in China are local NNESTs (Pawan, 2017). Most students only receive instruction from NNESTs from elementary school to high school. Both students and instructors are heavily influenced by the traditional teacher-centered educational model in China. This model accentuates teachers’ authority and professional abilities and focuses on knowledge transmission (Yang and Zhang, 2015; Gao, 2017; Liu, 2021b). Teachers serve an important role as the source of information and keep track of students’ learning progress, while students are seen as passive recipients of knowledge. It is common for teachers to give lectures for the majority of the class and students to attentively listen and take notes (Emaliana, 2017). In addition, NNESTs generally have a higher academic degree and more teaching experience. They are familiar with a variety of second language learning theories and students’ psychology. Therefore, they often observe students’ progress and potential to help students in their learning process and advancement (Xu, 2014). In contrast, NESTs rarely talk about these essential qualities. They give greater importance to affective characteristics such as being patient, caring, flexible, and sensitive to students. This could be explained by the fact that NESTs are usually recruited from inner-circle countries and have received Western education. For instance, the NESTs participants received their education in the USA. Western countries such as the USA have slowly moved from teacher-centered education to student-centered education (Bernard et al., 2019). NESTs are likely to have benefited from student-centered education and applied it when teaching Chinese EFL students. This educational approach focuses on being flexible in delivering content, encouraging student participation, and meeting learners’ needs (Wright, 2011). Subsequently, NESTs have come to value personal and affective qualities (e.g., fairness and caring) that fit in with the student-centered educational model.

Research has demonstrated that both prerequisite qualities (e.g., knowledge and professionalism) and affective qualities are both critical to successful teaching (Al-Seghayer, 2017; Stronge, 2018; Li et al., 2022). As a result, rather than attempting to decide who has a better understanding of the qualities of an effective teacher, it is crucial that teachers and students gain a systematic understanding of the functions of the different qualities and strive to achieve a better alignment. Nevertheless, the largely uninformed opinions of the different groups imply that participants tend to place too much importance on certain qualities while disregarding others. For instance, NNESTs and students tend to focus on prerequisite qualities, while overlooking affective qualities. On the other hand, NESTs prioritize affective qualities over prerequisite qualities. These discrepancies in the perceptions of effective teaching qualities among the participants may have been caused by their diverse linguistic, educational, and cultural backgrounds, which additionally contribute to stereotypical positive and negative characteristics of NESTs and NNESTs.

## Conclusion

This investigation examined the perspectives of students, NESTs, and NNESTs on teaching effectiveness for a comprehensive understanding of their similarities and disparities. Semi-structured interviews were conducted for teachers and students and thematic analysis was employed for the data analysis. Overall, this study illustrates the misalignments between students’, NESTs’, and NNESTs’ perceptions of effective teachers’ characteristics in the Chinese EFL context and discussed the underlying cultural, educational, and individual factors that contribute to the misalignments. The results demonstrated that Chinese students and NNESTs have more resemblance in their opinion of effective teacher characteristics than NESTs. Both prioritize knowledge, teaching skills, and professionalism. In comparison, NESTs place more emphasis on individual qualities and student engagement such as being caring and flexible. These discrepancies could have been caused by differences in the participants’ beliefs of educational models, given that NNESTs and students are familiar with the teacher-centered model while NESTs are more accustomed to the student-centered model. In conclusion, this research showed the misalignments between students, NESTs, and NNESTs in terms of their views on effective teacher characteristics in the Chinese EFL contexts, along with the cultural, educational, and individual aspects contributing to these differences.

The results of this study might have important implications for educators, learners, and university administrators. These implications could help enhance the effectiveness of teachers, and improve the collaboration between NESTs and NNESTs. The study has found that NEST, NNESTs, and students have different prioritizations on effective teachers’ characteristics, which are likely to result from their different cultural, linguistic, and educational backgrounds. Research has shown that certain negative or neglected qualities of NESTs and NNESTs can be developed through training (Huang, 2017). Therefore, university administrators could offer training and education to NESTs and NNESTs to understand the complementary nature of various effective teacher characteristics in teaching, allowing them to bolster weaker areas and help improve instruction. For example, NNESTs could cultivate their affective qualities and be more caring, respectful, and fair to students, and learn about student-centered teaching methodologies (Wang and Fang, 2020). NESTs, in the meantime, could put more effort into understanding the Chinese culture and education system, adjusting their teaching materials and activities as needed, and making sure they are thoroughly prepared for class. NESTs and NNESTs could collaborate and teach together, as research has shown that co-teaching can result in the exchange of knowledge between teachers, and providing greater attention to learners’ needs (Gbenakpon, 2018; Rao and Yu, 2021; Sanders-Smith and Dávila, 2021). It also supports the professional growth of teachers, as they can improve their teaching practices by observing and learning from colleagues, as well as through exchanging practical teaching knowledge and mutual emotional support through a conducive teaching and learning environment (Zhang et al., 2023).

The study has several limitations. This research was conducted in a Chinese EFL setting, making the findings possibly limited in their generalizability to other ELT contexts. The sampling may also lack generalizability to non-English majors with various proficiency levels since the participants were all English majors from universities in China. Additionally, due to the impact of Covid-19, the scale of the study had to be reduced and all the NESTs participants were from the USA. Qualitative research methods such as class observation were also excluded due to limited access to the university campus. Future studies are essential to reflect the multifaceted and dynamic identity of NESTs and NNESTs by recruiting participants with diverse linguistic and cultural backgrounds. Lastly, this study utilizes a geopolitical contrast between the two groups and regards English teachers from Inner Circle countries (Kachru et al., 2006) as NESTs, which may not be applicable in other contexts.

Another possible limitation is concerned with the trustworthiness of the study. Trustworthiness refers to the degree of confidence in methods, data, and interpretation used to conduct high-quality research. “Trustworthiness or truth value of qualitative research and transparency of the conduct of the study is crucial to the usefulness and integrity of the findings” (Connelly, 2016). Clarke and Braun et al. (2017) divided the methods of providing the trustworthiness of thematic analysis (TA) into two broad types: ‘Small q’ and ‘Big Q’. The ‘Big Q’ is the utilization of techniques and instruments in the qualitative paradigm. The Big Q approach to TA works within a qualitative paradigm. It is theoretically independent and flexible, and the processes of creating codes and themes are organic. In the “Big Q” TA (e.g., the reflective thematic analysis), the analysis is more akin to a creative process than a technical process, as the researcher uses his own involvement, abilities, and analytical experiences in the analysis. Therefore, the reflective thematic analysis is more concerned with quality-assurance strategies such as a deeper engagement, and a thorough and systematic approach, than the accuracy of coding. These quality-assurance strategies were implemented to safeguard the trustworthiness of the current study. Although the student and teacher participants in four mid-tier universities may not reflect the diversity of all students and teachers. It is hoped that the findings can have implications in other similar settings. Further research is needed to address the limitations of this study. Further studies could explore how NESTs, NNESTs, and students can be better aligned in teaching practices so that they can improve their teaching toward high levels of effectiveness.

## Data availability statement

The raw data supporting the conclusions of this article will be made available by the authors, without undue reservation.

## Ethics statement

The studies involving human participants were reviewed and approved by the Human Ethics Committee of The University of Auckland, New Zealand. The patients/participants provided their written informed consent to participate in this study.

## Author contributions

LD and LZ conceived and designed the study. LD collected and analyzed the data and drafted the manuscript. LZ finalized it for submission as the corresponding author. All authors contributed to the article and approved the submitted version.

## Conflict of interest

The authors declare that the research was conducted in the absence of any commercial or financial relationships that could be construed as a potential conflict of interest.

## Publisher’s note

All claims expressed in this article are solely those of the authors and do not necessarily represent those of their affiliated organizations, or those of the publisher, the editors and the reviewers. Any product that may be evaluated in this article, or claim that may be made by its manufacturer, is not guaranteed or endorsed by the publisher.
