# Supplementary material for: Exploring native and non-native English speaker teachers’ perceptions of English teacher qualities and their students’ responses
Source: Front Psychol. 2023 Aug 15;14:1175379. doi: 10.3389/fpsyg.2023.1175379 (PMC10464833; doi:10.3389/fpsyg.2023.1175379)
Supplement: Supplementary file 1 [file Table_1.docx]

**Appendix A**

***Codebook for Thematic Analysis of Students’ Interviews: Themes, Codes, Descriptions, and Examples***

| **Theme** | **Code** | **Description and Example** |
| --- | --- | --- |
| Effective Teaching | Effective Teachers’ Qualities and Behaviors | Referring to teachers’ qualities and behaviors that are regarded by learners to have a positive effect on learning outcomes.  *e.g., Firstly, he should have a high level of professionalism. Secondly, he should be responsible. (Jennifer-FS)* |
|  | Expectation of Teaching | Learners’ idealized teaching form and environment.  *e.g., The expectations were that teachers would be able to show us more about the Western world, different from the Chinese model (Robert-MS)* |
|  | Suggestions on Improving Teaching Effectiveness | Learners’ suggestions on improving the teaching effectiveness of English classes.  *e.g., I think this is something that needs to be improved. I think I would like to learn more about culture from NESTs (Isabella-FS)* |
| Differences  Between NESTs and NNESTs | Positive Characteristics of NNESTs | Learners’ perceptions on the positive characteristics of NNESTs.  *e.g., The positive characteristics of NNESTs are that they are more effective at communicating with you. (Ryan-MS)* |
|  | Negative Characteristics of NNESTs | Learners’ perceptions on the negative characteristics of NNESTs.  *e.g., A NNEST puts too much emphasis on written exercises and test preparation. (Ryan-MS)* |
|  | Positive Characteristics of NESTs | Learners’ perceptions of the positive characteristics of NESTs.  *e.g., I think their pronunciation is generally better than NNESTs (Jane-FS)* |
|  | Negative Characteristics of NESTs | Learners’ perceptions on the positive characteristics of NNESTs.  *e.g., The disadvantage is that sometimes he lacks a syllabus or does not follow a syllabus. (Jane-FS)* |
|  | Views on Accent | Learners’ perceptions of different English accents.  *e.g., I think the British accent is quite nice and sounds very elegant. (Roman-MS)* |
|  | Nativeness | Learner’s perceptions on the effect of nativeness in English teaching.  *e.g., I don't think it's a necessity. Many native English speakers may be a little bit better at spoken English, but they are not as good at grammar as someone who specializes in English grammar. (Charlotte-FS)* |
|  | Learning Experience with NESTs and NNESTs | The number of NESTs and NNESTs that the participant had at university.  *e.g., There were probably only three NESTs, and the rest were NNESTs. (Robert-MS)* |

**Appendix B**

***Codebook for Thematic Analysis of Teachers’ Interviews: Themes, Codes, Descriptions, and Examples***

| **Theme** | **Code** | **Description and Example** | |
| --- | --- | --- | --- |
| Differences between NESTs and NNESTs | Positive Characteristics of NESTs | Teachers’ perceptions of the positive characteristics of NESTs.  *e.g., The positive characteristics (of NESTs) are that their language is more authentic, and they are also more flexible in their teaching methods. (Lily-FNNEST)* |  |
|  | Negative Characteristics of NESTs | Teachers’ perceptions of the negative characteristics of NESTs.  *e.g., Their biggest problem may be the classroom organization, which they are not as used to and comfortable with as the Chinese teacher. (John—MNNEST)* |  |
|  | Positive Characteristics of NNESTs | Teachers’ perceptions of the positive characteristics of NNESTs.  *e.g.,* A Chinese teacher is more familiar with the learning environment of Chinese students, their learning process, the characteristics of their English learning, and the students themselves. (Lily-FNNEST) |  |
|  | Negative Characteristics of NNESTs | Teachers’ perceptions of the negative characteristics of NNESTs.  *e.g., Some of the drawbacks that they have is that they sometimes have some pronunciation problems or some grammatical problems as well. (Claire-FNEST)* |  |
| Teaching Beliefs | Formation of Teaching Beliefs | The foundation of teachers’ beliefs in teaching students.  *e.g., I had to learn about Chinese culture and how students operate in classrooms here that are different from other countries before I could really be effective as a teacher. (Claire-FNEST)* |  |
|  | Effective Teachers' Qualities | Referring to teachers’ qualities and behaviors that are regarded by teaching to have a positive effect on teaching outcomes.  *e.g., I think it is also important for teachers to have a wide range of knowledge and life experience. (David-MNNEST)* |  |
|  | Nativeness | Teachers’ perceptions on the role of nativeness in English teaching.  *e.g., I don't think so. After all, the vast majority of people teaching foreign languages in China are non-native English speakers. (Participant: Lily-FNNEST)* |  |
| Teachers’ Background Information | Nationality | Teachers’ nationality.  *e.g.,* I am American. *(Claire-FNEST)* | |
|  | Educational Background | Teachers’ educational background.  *e.g.,* My highest degree was a PhD in translation. (Lily-FNNEST) | |
|  | First Language and Second Language | Teachers’ first language and second language.  *e.g., Chinese. I studied English and a little bit of Japanese. (Nora—FNNEST)* | |
|  | Teaching Experience | Teachers’ teaching experience.  *e.g., I taught in China for 3.5 years*. (Lucy-FNEST) | |
